# Roles of extracellular vesicles in the pathogenesis of chronic myeloid leukemia

**DOI:** 10.1007/s13105-026-01184-0

**Published:** 2026-04-17

**Authors:** Tereza Hrdinova, Helena Kupcova Skalnikova

**Affiliations:** 1 Laboratory of Proteomics, Institute of Biochemistry and Experimental Oncology, First Faculty of Medicine, Charles University, U Nemocnice 5, Prague, 128 53 Czech Republic; 2 BIOCEV, First Faculty of Medicine, Charles University, Prumyslova 595, Vestec, 252 50 Czech Republic

**Keywords:** Chronic myeloid leukemia, Extracellular vesicle, Bone marrow, Microenvironment, Marker, Tyrosine kinase inhibitor, Resistance

## Abstract

Extracellular vesicles (EVs) are lipid bilayer-enclosed particles released by cells into the extracellular space. They are produced by the majority of cell types under both physiological and pathological conditions. EVs mediate intercellular communication by delivering bioactive molecules - such as RNAs, adhesion molecules, signaling mediators, and antigen-presenting molecules - to recipient cells, thereby modulating their phenotype and function. A growing body of evidence highlights the involvement of EVs in the pathophysiology of leukemia, including their roles in remodeling the bone marrow microenvironment, promoting angiogenesis, enabling immune evasion, regulating apoptosis, and contributing to drug resistance. Beyond their functional roles, EVs are emerging as promising diagnostic and prognostic biomarkers, as well as potential drug delivery vehicles. This review first outlines key aspects of chronic myeloid leukemia and the bone marrow niche, and then focuses on the current understanding of how EVs contribute to the transformation of a normal bone marrow environment into a leukemic one. Finally, we discuss the potential applications of EVs as biomarkers and therapeutic delivery systems in chronic myeloid leukemia.

## Key Points

Extracellular vesicles (EVs) participate in intercellular communication.

CML cell derived EVs contribute to the remodeling of bone marrow microenvironment.

EVs participate in spreading of imatinib resistance among cells.

EVs carry diagnostic/prognostic markers and can be used as drug delivery vehicles.

## Introduction

### Chronic myeloid leukemia

Chronic myeloid leukemia (CML) is a clonal malignant myeloproliferative disease characterized by the presence of Ph chromosome, which results from reciprocal translocation between chromosomes 9 and 22 [1, 2]. This translocation leads to a fusion of a part of the Abelson leukemia virus oncogene (ABL) normally located on chromosome 9 with a part of the breakpoint cluster region gene (BCR) normally located on chromosome 22 [3, 4]. The resulting *BCR::ABL1* fusion gene leads to production of constitutively active BCR-ABL kinase.

BCR-ABL kinase is responsible for pathogenesis of the disease due to aberrant oncogenic signaling which activates intracellular signaling pathways leading to cell proliferation, inhibition of apoptosis and alteration in cellular adhesion [5–9]. In addition, BCR-ABL kinase stimulates production of reactive oxygen species (ROS) which contribute to genomic instability and disease progression [10]. Progression of CML is further influenced by multiple factors including genomic instability, accumulation of additional mutations and chromosomal aberrations, differentiation arrest, and metabolic changes [11, 12]. CML progresses from chronic phase to accelerated and blast phase, which is characterized by differentiation arrest and accumulation of immature blasts in bone marrow (BM) and peripheral blood [13].

Development of targeted therapy using tyrosine kinase inhibitor (TKI) imatinib mesylate revolutionized the treatment of CML [14–16]. Imatinib dramatically improved the long term overall patient survival to more than 90%. Nonetheless, approximately 20% of patients develop resistance to the TKI therapy [17].

TKI resistance arises from two distinct mechanisms: BCR-ABL-dependent resistance, where point mutations or amplification of *BCR::ABL1* gene lead to the loss of imatinib efficacy, and, BCR-ABL-independent resistance, characterized by insufficient intracellular concentration of imatinib in CML cells, activation of alternative signaling pathways, or acquisition of additional genetic alternations [18, 19]. Moreover, leukemic stem cells (LSCs) remain a challenge in the treatment of CML because they are resistant to imatinib [20, 21]. Deeper understanding of molecular mechanisms driving leukemia progression and resistance may provide novel approaches in anti-CML therapy.

Increasing number of studies has demonstrated that leukemia cells communicate by direct cell-to-cell contact, secreted soluble factors (e.g. cytokines, chemokines), and also by release of extracellular vesicles (EVs) that mediate crosstalk between leukemia cells and bone marrow microenvironment affecting the growth, survival, proliferation, and drug resistance of leukemia cells [22–26].

### Extracellular vesicles

EVs are highly heterogenous membrane surrounded extracellular particles, unable to replicate, which are released from various cell types under both normal and disease-related conditions [27–29]. EVs differ in size, biogenesis mechanisms, release pathways, molecular composition, and morphology [27]. According to their biogenesis, EVs are typically classified into three distinct groups: exosomes, microvesicles, and apoptotic bodies [27]. Exosomes are the smallest type of EVs (generally smaller than 200 nm) that are formed in the endosomal compartment of cells and subsequently released into extracellular environment by fusion of multivesicular body membrane with the plasma membrane [30–33]. Microvesicles are generated from plasma membrane by outward budding, while apoptotic bodies are formed through blebbing of the cell membrane during apoptosis [34, 35]. However, due to challenges associated with identifying the precise vesicle biogenesis pathways, recent guidelines by the International Society for Extracellular Vesicles (ISEV) recommend using the general term EVs (or small EVs for particles with a diameter less than 200 nm and large EVs for particles with a diameter larger than 200 nm). The terms related to biogenesis, e.g., exosomes or microvesicles, should be used only with caution unless these vesicles are specifically separated and characterized [29, 36]. Therefore, in this review, the term EVs will be used, despite the term exosomes or others are frequently used by the authors of cited publications.

Initially, EVs and particularly exosomes were originally described to remove unnecessary proteins such as transferrin receptor from cells, however, nowadays they are considered as important mediators of short- and long-distance cell-to-cell communication [30–33]. Their role in intercellular communication stems from their ability to transport and deliver various molecules, including proteins, lipids, and nucleic acids (DNA, RNA, miRNA, lncRNAs), that reflect their cells of origin, to the recipient cells [37–44]. In some cases, EVs have been shown to carry whole mitochondria [45, 46]. Intercellular communication mediated by EVs affects numerous physiological and pathological processes [47–54]. During cancer development, EVs may be one of the factors actively and effectively reshaping the healthy microenvironment into a pro-tumorigenic one [55]. As reviewed here for CML, most studies have focused on small EVs, particularly those within the size range of exosomes. These vesicles have been shown to affect proliferation of CML cells, modify BM microenvironment, induce angiogenesis, and contribute to drug resistance transfer [25, 54, 56].

### Bone marrow microenvironment

BM is a primary tissue of adult hematopoiesis. It is located in central cavities of long and axial bones. BM is complex milieu composed by various specific cell populations, including hematopoietic stem cells (HSCs), mesenchymal stromal cells (MSCs), various types of vascular and sinusoidal endothelial cells, osteoblasts, osteoclasts, bone lining cells, macrophages, perivascular CXCL12-abundant reticular cells (CARs), neurons, and adipocytes. BM microenvironment is further formed by extracellular matrix and adipose tissue [57].

Normal HSCs reside within BM and are supported by specialized and spatially highly organized vascular and endosteal stem cell niches [58–60]. The BM niche maintains the normal hematopoiesis (HSC proliferation, survival, and self-renewal) by functional crosstalk between cells [61, 62]. Particularly bone marrow mesenchymal stromal cells (BM-MSCs) cells represent important component of bone marrow niche controlling maintenance and fate of HSCs and secreting various cytokines, growth factors and EVs [61, 63]. EVs derived from MSCs modulate normal hematopoiesis through diverse mechanisms, including regulation of quiescence, differentiation, and trafficking. Murine MSCs-derived EVs disrupt HSCs quiescence and enhance expansion of murine myeloid progenitors [61]. In addition to increasing the viability and inducing less differentiated state, EVs can alter expression profile of CD34 + from umbilical cord blood [64]. MSCs-derived EVs increase G-CSF induced HSCs and progenitor cell mobilization [65]. EVs derived from MSCs transfer proangiogenic miRNA molecules to endothelial cells and increase formation of tube-like structures [66]. EVs derived from other cells also affect HSCs. Megakaryocytic EVs stimulate differentiation of HSCs and progenitor cells into megakaryocytes [67]. Finally, changes in microenvironmental conditions such as oxygen concentration can alter HSCs function. Hypoxia-preconditioned MSCs-derived EVs increase self-renewal capacity, quiescence and clonogenic potential of umbilical cord blood hematopoietic stem cells [68].

In leukemia development, complex genetic mutations transform HSCs or progenitor cells into LSCs that retain self-renewal capacity, undergo uncontrolled proliferation, and due to the block in differentiation, leukemic blasts are produced [69–72]. LSCs occupy the identical bone marrow niche as normal HSCs, thus gaining advantages from the supporting microenvironment. Moreover, LSCs have the ability to remodel the BM niche to create an environment that is less favorable for normal HSCs. Such modified BM niche is more beneficial for LSCs growth and dissemination, so they become the predominant cell population in BM [73–75].

BM microenvironment provides crucial signaling crosstalk between cells playing an important role in leukemia progression. This communication takes place via direct cell-to-cell contact, secretion of regulatory molecules including growth factors and cytokines, or through other mechanisms including EVs [76–81].

In the experimental in vitro conditions, intercellular interactions in BM microenvironment are mostly modeled using CML cell lines such as LAMA84 and K562, which were derived from patients with chronic myeloid leukemia in blast crisis and which are widely used in CML research [82, 83]. To imitate the interactions between CML cells with stromal cells, the HS-5 bone marrow stromal cells or BM-MSCs have been used [84, 85]. Similarly, studies investigating the effects of CML-derived EVs on adhesion to endothelial cells and angiogenesis often use human umbilical vein endothelial cells (HUVECs) [86, 87]. Primary isolates of macrophages and T cells have been utilized to study CML EVs interaction with immune cells [23, 24]. Although these cellular models considerably simplify the complex in vivo microenvironment and organism-level interactions, they provide valuable initial insights into intercellular interactions under controlled conditions.

## Roles of EVs in CML

**Fig. 1:**
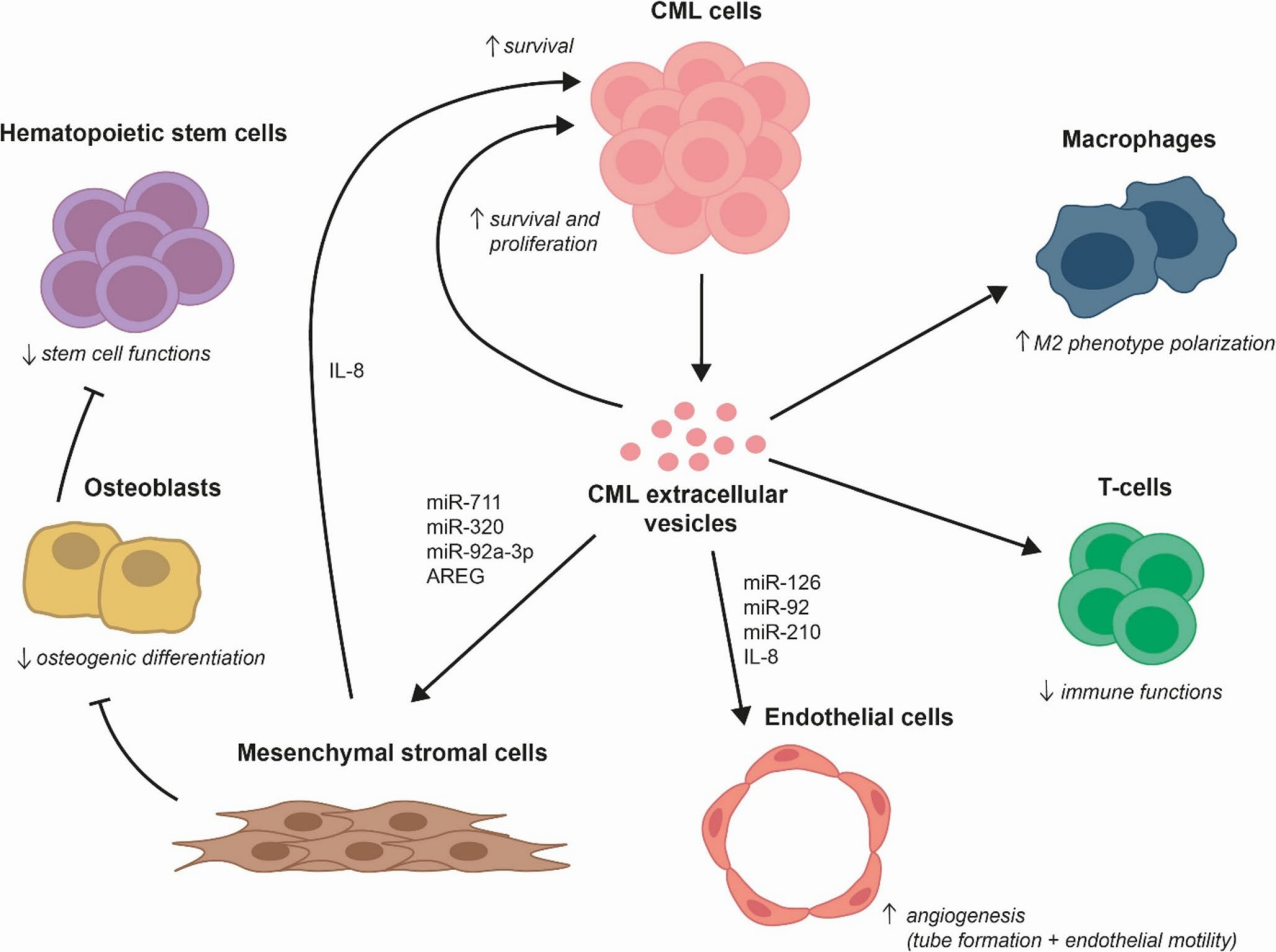
Effects of chronic myeloid leukemia cell-derived extracellular vesicles on bone marrow microenvironment

EVs derived from CML cells have been demonstrated to be capable of remodeling of BM niche into leukemia favorable microenvironment, in particular by altering cellular adhesion, osteogenesis, and angiogenesis. CML-derived EVs also exhibited direct effects on CML cell proliferation, immune escape, and resistance to cell death (Fig. 1). In the following paragraphs, we review current understanding of EVs functions in CML.

CML-derived extracellular vesicles (EVs) transfer miRNA, mRNA, and proteins to bone marrow stromal cells (MSCs), endothelial, and immune cells, remodeling the microenvironment. EVs directly support CML survival and proliferation and indirectly promote it by enhancing MSCs adhesion, IL-8 production, inhibiting MSCs osteogenesis, boosting endothelial angiogenesis, and impairing host immune system via T-cell suppression and M2 macrophage polarization. These changes drive CML progression.

### Adhesion

Cell adhesion is essential for regulation of fundamental cellular processes, such as survival, proliferation, and migration. Adhesive interactions maintain homeostasis within BM niche [88]. In leukemic BM, adhesion of leukemic cells to stroma promote survival of malignant cells [89]. Disruption of adhesive interactions can facilitate migration of malignant cells and the disease dissemination.

Several in vitro studies examined effects of CML-derived EVs on adhesion. Corrado et al. used a model of chronic myeloid leukemia cell line LAMA84 and stromal cell line HS-5. They discovered that LAMA84-derived EVs induced expression of interleukin 8 (IL-8) in HS-5 cells, and IL-8 released into the extracellular space supported adhesion of LAMA84 cells to stromal cells [89]. Subsequently, it was found that EVs from LAMA84 cells as well as from leukemic cells isolated form blood of newly diagnosed CML patients contained amphiregulin (AREG) [90]. When CML-derived EVs were added to HS-5 stromal cells, AREG activated epidermal growth factor receptor (EGFR) signaling in stromal cells resulting in increased expression of transcription factor SNAIL and its targets IL-8 and matrix metalloproteinase-9 (MMP-9) [90]. In addition, the LAMA84-derived EVs stimulated expression of Annexin A2 in HS-5 stromal cells. Results from both studies by Corrado et al. demonstrate an EVs-mediated crosstalk between leukemia cells and stromal cells, leading to activation of EGFR signaling and increased expression of annexin A2 in stromal cells, thereby sustaining the adhesion, growth, survival and invasiveness of CML cells [89, 90].

Another study demonstrated that CML-derived EVs can decrease adhesion capabilities of MSCs. Jiang et al. treated BM-MSCs by EVs derived from K562 cells. Such leukemic EVs were able to transfer miR-711 into BM-MSCs, which led to deregulation of expression of the adhesion molecule CD44 in BM-MSCs and to their decreased adhesion [91].

The miRNA profiling of CML cells and CML-derived EVs revealed enrichment of miRNAs within EVs affecting adhesion of CML cells to endothelial cells [92, 93]. Feng et al. compared miRNAs levels between K562 cells and K562 EVs and identified 49 miRNAs, including miR-711, to be abundant in EVs [93]. Bioinformatic analysis of such miRNA function by Gene Ontology and KEGG pathways revealed their association with cellular adhesion. Functional in vitro assay confirmed decreased adhesion of K562 cells to endothelial HUVEC cells after pretreatment of HUVEC cells with K562-derived EVs [93]. In an additional study focusing on miRNA in leukemia cell EVs, Taverna et al. identified 18 miRNAs exclusively present in EVs and 106 differentially expressed miRNAs including miR-126 between LAMA84 cells and LAMA84 EVs [92]. The miR-126 enriched LAMA84-derived EVs were capable of down-regulation of expression of CXCL12 chemokine and vascular cell adhesion molecule 1 (VCAM-1). Decreased adhesion of LAMA84 cells to endothelial monolayer following prolonged exposure to LAMA84-derived EVs facilitated the migration of leukemic cells through endothelial monolayer and may represent one of the mechanisms contributing to CML progression [92]. In addition, EV containing miR-126 from endothelial cells were described to modulate quiescence and proliferation of CML LSCs [94].

### Osteogenesis

Osteogenic differentiation of MSCs is important for bone tissue regeneration and for homeostasis of BM microenvironment. There is a correlation between the number of osteoblasts and the number of HSCs [95]. Osteoblasts regulate normal HSCs self-renewal and their elimination accelerates the development of leukemia [96].

Inhibition of osteogenesis by CML-derived EVs was described by Gao et al. in their investigation of molecular and functional changes in BM-MSCs after exposure to EVs derived from K562 cells [97]. K562 derived EVs were endocytosed by surrounding BM-MCSs leading to a decreased capacity of osteogenic differentiation of BM-MSCs in vitro as was demonstrated by alkaline phosphatase and Alizarin Red staining. These results were further confirmed in vivo by intravenous injection of leukemia EVs into mice. Mice treated by leukemia EVs exhibited loss of whole bone volume and decreased frequency of HSCs indicating damage of BM niche. RNA sequencing identified miR-320 as a potential mediator of osteogenesis inhibition, possibly via downregulation of β-catenin [97].

### Effects of EVs on angiogenesis

Endothelial cells are key component of vascularized tissues including BM niche and play a role in formation of new blood vessels in normal and leukemic BM [98, 99]. Several studies clearly proved the proangiogenic effects of CML-derived EVs. Taverna et al. investigated the effects of CML-derived EVs on angiogenesis by incubating LAMA84-derived EVs with HUVEC cells, and subsequent quantifying of cytokines and adhesion molecules, as well as studying cellular adhesion and migration [56]. LAMA84-derived EVs stimulated neoangiogenesis by increasing HUVEC motility and tube formation via activation of mitogen-activated protein kinase (MAPK) signaling pathway [56]. Similar proangiogenic effects were demonstrated by EVs derived from K562 cells and from whole blood of two patients [56, 100, 101]. Further results revealed that Src kinase activation and transfer of pro-angiogenic miR-92a were responsible for HUVEC cell migration and tube formation [100, 102]. Data obtained in vitro were consistent with in vivo experiments conducted in nude mice subcutaneously implanted with synthetic extracellular matrix (Matrigel) plugs containing K562- and LAMA84-derived EVs [56, 100].

Hypoxia is characteristic for tumor microenvironment. Altered miRNA profiles have been identified in leukemic cells and EVs under hypoxic conditions. In K562 cells and their respective EVs, levels of certain miRNAs (miR-18b, miR-210) were increased under hypoxic conditions in contrast to normoxia. To determine vesicle communication between hypoxic CML cells and endothelial cells, EVs derived from K562 cells cultured under hypoxic condition were incubated with HUVEC cells. EVs derived from hypoxic K562 cells enhanced the tube formation by HUVEC cells via miR-210, which is known regulator of hypoxia [103, 104].

On the other hand, composition of EVs derived from CML cell lines could be altered towards antiangiogenic vesicle properties by natural antitumor compound curcumin. Curcumin is a hydrophobic polyphenol isolated from rhizome of *Curcuma longa*, which exerts anti‑inflammatory, antioxidant, neuroprotective and anti-cancer properties. Curcumin is widely used in Asian traditional medicine and has shown potential in several clinical trials in cancer treatment [105]. EVs derived from cell lines K562 and LAMA84 treated with curcumin were enriched in miR-21 and were able to transfer miR-21 into HUVEC cells. The miR-21 in HUVEC cells inhibited expression of small GTPase RhoB and myristoylated alanine rich C-kinase substrate (MARCKS) protein which was followed by decresed motility of HUVEC cells and inhibition of tube formation. These data demonstrate antingiogenic potential of EVs from leukemic cell lines treated with curcumin [106].

### Effects of EVs on immune cells and immunosuppression

Effector cells of the immune system are capable to eliminate tumor (leukemia) cells. However, as leukemia develops and progresses it suppresses and evades the immune system control. Such immunosuppressive condition can be caused by various factors including suppressive immune cells, receptors, cytokines, and EVs released from leukemia cells [107].

EVs derived from serum of CML patients have been shown to induce conversion of healthy donor monocytes into monocytic myeloid-derived suppressor cells (M-MDSCs). These M-MDSCs demonstrated immunosuppressive activity by reducing proliferation of T-cells [108].

Further studies elucidated immunomodulatory and tumor-promoting effects of K562 derived EVs on BM-MSCs, macrophages and T-cells [23, 24]. In macrophages, treatment by K562-derived EVs increased expression of anti-inflammatory cytokines IL-10 and tumor necrosis factor alpha (TNF-α), and reduced levels of ROS, suggesting polarization of macrophages into M2 phenotype. This phenotype is known in hematological malignances to contribute to immunosuppression, angiogenesis, and tumor growth [23]. In T-cells, K562-derived EVs induced overexpression of genes involved in inhibition of T-cells (*NQO1*, *PDCD1*, and *FOXP3*) and downregulation of genes involved in T-cell activation (*CD3D* and *NFATC3*) [24]. Additionally, K562-derived EVs stimulated IL-10 and IL-6 mRNAs expression in T-cells and increased secretion of IL-6, IL-10, and IL-17 cytokines from T-cells [24].

Moreover, nitric oxide (NO) has been recognized as signaling molecule affected in immune cells by CML-derived EVs. NO has complex roles in cancer, such as triggering of vasodilatation and stimulation of angiogenesis, while it also possesses cytotoxic and immunomodulatory properties. The effects mostly depend on time and dose of NO released, cell type and redox milieu [109, 110]. In both BM-MSCs and T-cells, treatment with CML derived EVs induced production of NO, while in macrophages led to reduction of NO synthesis [23, 24].

Altogether, changes induced in immune and stromal cells by CML-derived EVs seem to contribute to formation of an immunosuppressive microenvironment in BM niche that promotes leukemia progression [23].

### Adipogenesis

Adipogenesis is a process of development and accumulation of adipocytes in the form of adipose tissue. Patients in advanced stages of cancer suffer from cancer associated cachexia, characterized by body weight loss, particularly muscle mass and fat mass loss [111]. Adipose-derived MSCs represent a subset of MSCs capable of extensive proliferation and differentiation into various cell lineages, including adipocytes [112].

EVs derived from lung cancer, gastric cancer and CML have been found to modulate adipogenesis [113]. In an in vivo experiment, mice intravenously injected with K562-derived EVs exhibited body weight drop and fat loss. To elucidate the effect of K562-derived EVs on adipogenesis, such EVs were incubated with adipose tissue-derived MSCs. K562-derived EVs were taken up by adipose tissue-derived MSCs and inhibited adipogenic differentiation of MSCs [114]. RNA sequencing revealed miR-92a‐3p to be highly abundant in CML cells and CML-derived EVs. Such EVs transferred miR92a‐3p to adipose-derived MSCs, resulting in decreased expression of mRNA for adipogenic transcription factors C/EBPα and adipocyte-specific markers [114]. Nonetheless, additional studies are required to elucidate possible effects of leukemic EVs on bone marrow MSC differentiation to adipocytes and adipogenesis.

### Effects of EVs on proliferation and survival of CML cells

Beside the effects of EVs on other cells in the tumor microenvironment, the CML-derived EVs can affect the CML cells themselves, via an autocrine mechanism. EVs derived from LAMA84 cells were shown to promote LAMA84 cell proliferation in a dose-dependent manner [54]. To better understand molecular processes allowing leukemia cell proliferation and escaping from apoptotic death, Raimondo et al. studied vesicle involvement in pro- and anti-apoptotic balance with focus on one of the key factors controlling cell survival and death, transforming growth factor beta 1(TGF-β1) [54, 115]. LAMA84-derived EVs were shown to carry TGF-β1 and were able to activate extracellular signal-regulated kinase (ERK), protein kinase B (AKT), and nuclear factor kappa-light-chain-enhancer of activated B cells (NF-κB) in recipient LAMA84 cells [54]. Expression of anti-apoptotic proteins [survivin, B-cell lymphoma-extra large (BCL-xL), and BCL-2-like protein 2 (BCL-W)] and mRNAs was increased and expression of pro-apoptotic proteins [BCL2 associated agonist of cell death (BAD), bcl-2-like protein 4 (BAX) and BCL2 binding component 3 (PUMA)] and their respective mRNAs was reduced in recipient LAMA84 cells after treatment with EVs [54].

A molecule identified to promote survival of cancer cells including CML cells is IL-8 [116–118]. Activation of AKT kinase was suggested to mediate the IL-8 effect on LAMA84 cell survival [89]. The involvement of IL-8 in CML cell survival and tumor growth was confirmed also in vivo in xenograft mouse model, where mice treated with IL-8 developed larger tumors [89].

In addition, leukemic cells have been shown to decrease cellular levels of growth suppressive miRNAs (e.g. miR-320) by their selective sorting into EVs. Differential enrichment of miRNAs in EVs compared to cells of their origin was observed in K562 as well as LAMA84 cells and was further confirmed in clinical CML samples [119].

**Fig. 2:**
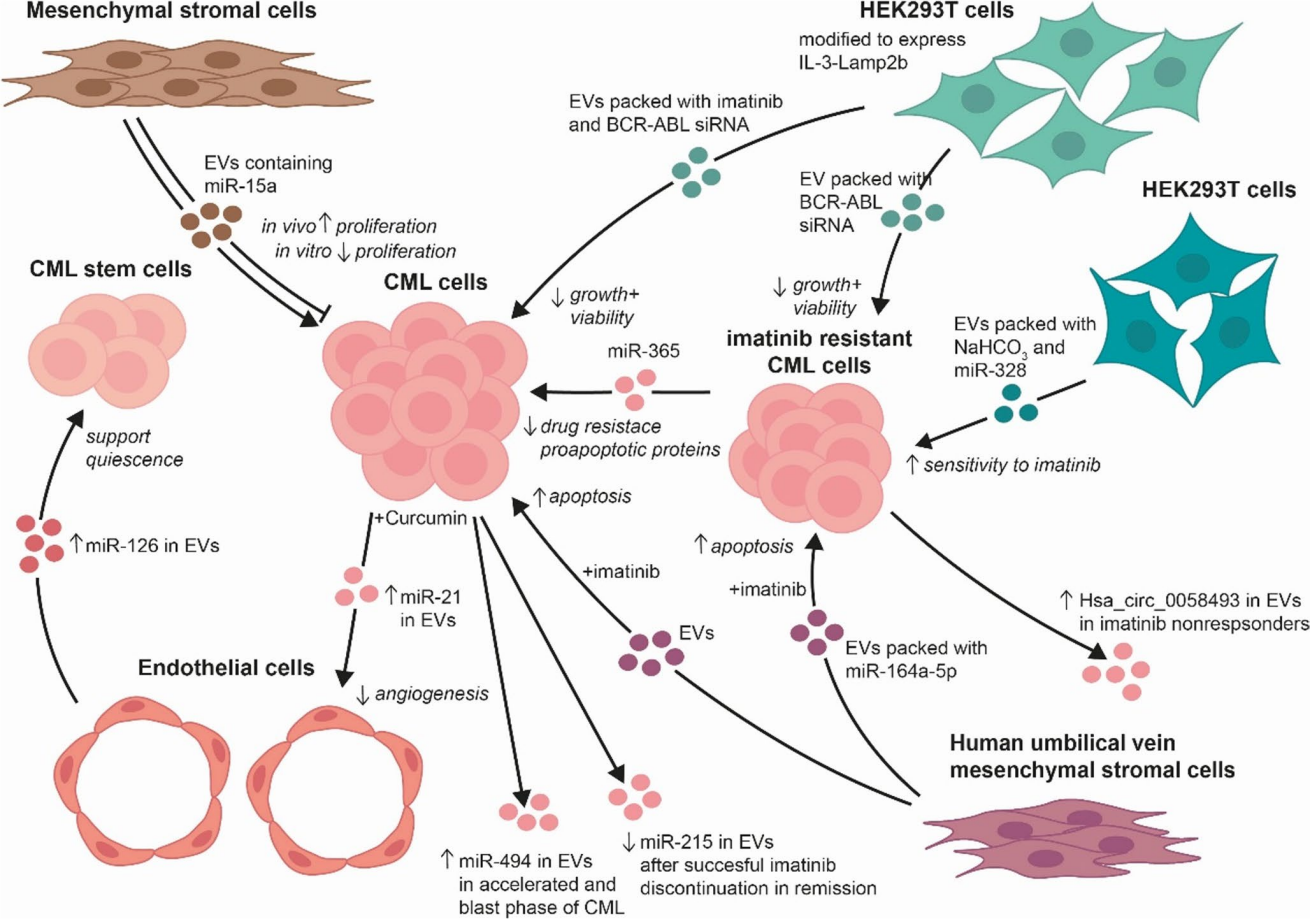
Chronic myeloid leukemia cells are affected by extracellular vesicles released by other cells

CML cell survival and proliferation can be affected not only by the CML derived EVs, but also by EVs derived from stromal cells (Fig. 2). In vitro results demonstrated that human BM-MSC-derived EVs inhibited proliferation of CML cells and arrest them in G0/G1 phase of cell cycle via miR-15a [120]. However, opposite results were obtained in vivo using on BALB/c nu/nu mice. In such mouse model, BM-MSC EVs promoted proliferation and drug resistance, tumor growth and tumor incidence [120]. In the tumor xenograft model, higher concentrations of BM-MSC-derived EVs increased proliferation and decreased apoptosis of CML cells in the presence of imatinib. Expression of anti-apoptotic protein B-cell lymphoma 2 (BCL-2) was increased and caspase-3 expression decreased in CML cells after treatment with higher doses of BM-MSC EVs in combination with imatinib [120]. These results show that BM-MSC-derived EVs may decrease sensitivity of CML cells to imatinib.

EVs from various cells (e.g., MSCs, endothelial) modulate CML cell viability, proliferation, apoptosis, quiescence of stem cells, and imatinib resistance via bioactive molecules. EVs from imatinib-resistant CML cells can transfer drug resistance to sensitive cells. Therapeutically modified EVs (e.g., from HEK293T or MSCs) sensitized resistant CML cells to imatinib and reduced viability. EV from curcumin-treated CML attenuated angiogenesis. EVs also serve as prognostic carriers: low levels of certain miRNAs marked successful imatinib cessation, while elevated miRNAs/circRNAs indicated advanced disease or non-response.

### BCR-ABL1 and drug resistance transfer via EVs

K562-derived EVs have been shown to contain BCR-ABL fusion protein, *BCR::ABL1* mRNA, and also *BCR::ABL1* DNA [121–124]. To investigate whether EVs may participate in the propagation of *BCR::ABL1* DNA to other cells, Cai et al. examined the transfer of *BCR::ABL1* gene [122]. This study demonstrated that EVs from K562 cell could transfer *BCR::ABL1* DNA to recipient HEK293T cells or neutrophils, with detectable expression of BCR-ABL protein in the recipient cells after 24 h of incubation [122]. To further demonstrate pathological significance of the transferred *BCR::ABL1* DNA, EVs derived from K562 cells carrying *BCR::ABL1* DNA were injected into the tail vein of immunodeficient mice, which subsequently developed CML-like disease symptoms such as fever, weakness, and splenomegaly [121, 122].

A growing number of studies highlight the role of EVs in transferring drug resistance among malignant cells. [125] Drug resistance is a significant challenge in the treatment of CML and main cause of therapy failure [126]. To elucidate whether EVs from imatinib-resistant cells are internalized by imatinib-sensitive cells, fluorescently labeled EVs derived from imatinib resistant K562 cells were incubated with sensitive K562 cells for 48 h. Confocal microscopy confirmed the internalization of these EVs [25]. Internalization of EVs derived from resistant CML cells to sensitive cells was later confirmed by other studies [127, 128]. To further clarify whether EVs derived from imatinib-resistant K562 cells are able to induce imatinib resistance in sensitive cells, Min et al. treated sensitive cells by EVs derived from imatinib-resistant cells and monitored cytotoxicity and apoptosis [25]. Upon treatment, originally sensitive cells showed reduced sensitivity to imatinib and decreased imatinib-induced apoptosis, indicating that EVs derived from imatinib-resistant cells transfer drug resistance trait to imatinib-sensitive cells [25]. To uncover the molecular mechanism underlying this phenomenon, miRNA analysis was performed. The most upregulated miRNA in EVs derived from imatinib-resistant cells was miR-365. Treatment of imatinib-sensitive K562 cells with these EVs resulted in elevated miR-365 levels in recipient cells, which reduced the expression of pro-apoptotic proteins such as BAX and cleaved caspase-3 [25]. Additional study performed by Karabay et al. also compared miRNAs expression profiles between sensitive and resistant cells, their respective EVs and sensitive cells treated with EVs from imatinib-resistant cells [129]. The authors found that miR-99a-5p and miR-125b-5p were increased, whereas miR-210-3p and miR-193b-3p were decreased in imatinib-resistant cells and their EVs. Bioinformatic analysis of the interacting genes and proteins of the discovered miRNAs identified several genes associated with imatinib resistance, including mTOR, STAT3, MCL1, LAMC1, KRAS [129]. In addition, higher expression of MDR1 and MCL1 was found in EVs from imatinib resistant K562 cells as well as in sensitive cells treated with EVs derived from imatinib-resistant cells. MDR1 is a predicted mRNA target of miR-193b-3p which was decreased in resistant cells and their EVs. Results from this study also suggest transport of MDR1 to sensitive cells via EVs from resistant leukemia cells [129]. Levels of another miRNA miR-629-5p were increased in K562 imatinib-resistant cells and EVs in contrast to findings reported by Karabay et al. [128–130]. EVs derived from imatinib-resistant cells transferred miR-629-5p to imatinib-sensitive cells. Overexpression of miR-629-5p in K562 reduced sensitivity of these cells to imatinib. The predicted target of this miRNA is SENP2, whose expression is diminished in K562 imatinib-resistant cells and EVs. SENP2 encodes a protease processing SUMO precursor for functional protein used for posttranslational modifications of proteins by SUMOylation. Experimental knockdown of SENP2 led to increased cell proliferation and decreased sensitivity to imatinib. SENP2 could inhibit AKT phosphorylation, and treatment of cells with imatinib decreased levels of phosphorylated protein levels from PI3K/AKT/mTOR signaling pathway. Additionally, results from peripheral blood mononuclear cells of newly diagnosed patients responding to imatinib, as well as those who developed blast crisis confirmed elevated expression levels of miR-629-5p in nonresponding patients [128]. EVs derived from K562 cells resistant to imatinib are internalized by imatinib-sensitive K562 cells, which leads in the sensitive cells to significantly increased survival under imatinib treatment [127]. Proteomic analysis of EVs derived from imatinib-resistant K562 cells revealed that these EVs harbor membrane proteins interferon-induced transmembrane protein 3 (IFITM3), CD146, and CD36. [127]. Recently was shown that expression of IFITM3 is regulated by BCR-ABL kinase via its interaction with ubiquitin specific peptidase 28 (USP28). High levels of USP28 were found in patients with BCL-ABL dependent CML and in imatinib-resistant K562 cells. Knockdown of IFITM3 decreased cell survival and induced apoptosis in K562 and imatinib-resistant K562 [131].

Altogether, these studies well documented the capacity of EVs for in vitro transfer of chemoresistance from imatinib-resistant cells into sensitive cells. Signaling pathway AKT/mTOR is one of the candidates being affected in recipient cells after such EV internalization.

### CML-derived EVs as prognostic factor and drug carriers

CML-derived EVs can be also viewed at as a source of prognostic, therapeutic, and diagnostic molecules as wells as potential therapeutic targets [132]. Due to their release into body fluids, EVs can be easily accessible from blood or other body fluids and serve as a liquid biopsy for discovery of biomarkers for non-invasive screening [41, 133]. In leukemia, analysis of EVs might be valuable in cases of complete remission when there are no leukemic cells in peripheral blood. Hallmarks of CML, i.e. *BCR-ABL1* gene, transcript, and protein have been used as biomarkers in CML cell lines and patient samples [124, 127, 134]. MiRNAs, other RNA types and proteins present in EVs may be associated with clinical symptoms and could be used as diagnostic markers as well.

To establish potential biomarker for patients who are able to discontinue imatinib treatment and maintain treatment-free remission, plasma and EVs were screened for miRNA using miRNA array. The miR-215 was shown as candidate biomarker for imatinib discontinuation as plasma and vesicle levels of miR-215 were significantly decreased in patients who successfully discontinued imatinib [135]. Imatinib discontinuation could be associated with musculoskeletal pain. To identify potential molecules involved in this symptom, vesicle miRNA profiling was performed. The miR-140-3p was identified to be enriched in EVs of patients with musculoskeletal pain [136]. On the other hand, miR-494 present in EVs could serve as a potential biomarker of poor response to TKI treatment and CML progression. Elevated levels of miR-494 were observed in patients with accelerated and blast phase CML [137]. In addition, miRNAs present in EVs could be promising therapeutic targets.

Circular RNAs are endogenous single-stranded covalently closed non-coding RNA molecules recognized as gene regulators with roles in development and progression of various cancers [138]. Zhong et al. studied circular RNA in plasma of patients responding and nonresponding to imatinib and in K562 cells sensitive and resistant to imatinib [139]. They identified circular RNA Hsa_circ_0058493 to be associated with imatinib resistance. In addition, Hsa_circ_0058493 levels were significantly increased in peripheral blood mononuclear cells (PBMCs) and EVs from imatinib nonresponding CML patients compared to imatinib responders. High levels of Hsa_circ_0058493 were associated with poor response to imatinib and such RNA has been suggested as a potential prognostic biomarker for CML [139]. Silencing of circular RNA Hsa_circ_0058493 represents potential target for elimination of resistant cells.

LncRNAs are single-stranded RNA molecules longer than 200 nucleotides that are acting mainly as regulators of gene expression by affecting stability and translation of mRNAs. Dysregulations of cellular lncRNA levels have been found in many cancers, including CML, where they were clearly shown to be associated with therapy resistance [140]. In EVs, the lncRNAs are much less explored. Wong et al. identified reduced levels of lncRNA LNC000093 in EVs from TKI-resistant K562 cells compared to sensitive cell EVs [141]. These authors show that reduced LNC000093 modulates the leukemic microenvironment towards a more pro-angiogenic one via regulating miR-675-5p levels. This was confirmed in an in vitro study, where EVs enriched in LNC000093 could reduce VEGF expression in BM-MSCs, thereby attenuating pro-angiogenic signaling [141].

To identify proteomic markers of imatinib resistance, EVs derived from imatinib-sensitive and imatinib-resistant cells were analyzed using label free quantification proteomic analysis. Surface proteins with markedly increased expression (CD146, IFITM3, and CD36) detected in EVs derived from imatinib-resistant K562 cells could be applicable as potential diagnostic markers of cells resistant to imatinib [127]. However, results from EVs isolated from plasma of CML patients responding and non-responding to imatinib revealed different overexpressed proteins as potential biomarkers of resistance to imatinib [142]. Thus, additional studies are required to elucidate candidate protein biomarkers of imatinib resistance.

EVs are attractive also as a drug delivery vehicle due to their biocompatibility and tumor specificity [143]. Bellavia et al. engineered EVs to deliver imatinib or BCR-ABL siRNA specifically into leukemic cells. In this study, HEK293T cells were modified to express vesicle lysosome-associated membrane protein 2 (Lamp2B) fused to IL-3 fragment. EVs expressing IL3-Lamp2B fusion protein were able to specifically target tumor cells in vitro and in vivo, as the CML and AML blasts highly express interleukin-3 receptor. EVs from imatinib treated HEK293T cells were able to reduce viability of CML cell in vitro and in vivo. HEK293T cell-derived EVs were found to be loaded with imatinib and to contain the IL-3-Lamp2b fusion protein [143]. Moreover, when such EVs were loaded with BCR-ABL siRNA, they reduced viability of leukemia cells *in vitro*, and suppressed tumor growth in vivo in mice subcutaneously inoculated with imatinib-resistant CML cells [143]. Additional study identified miR-328 to be significantly downregulated in imatinib-resistant cells by its increased degradation in lysosomes. The miR-328 was shown to downregulate expression of ABC transporter ATP-binding cassette super-family G member 2 (ABCG2), which is one of the transporters responsible for the drug efflux from cells. To overcome the imatinib resistance of K562 cells, EVs were alkalized and loaded by miR-328. Alkalized EVs from HEK293 cells containing miR-328 blocked lysosomal degradation of miR-328, decreased expression of ABCG2 transporter, and sensitized K562 cells to imatinib [144].

### MSC-derived EVs in treatment of CML

MSCs are multipotent cells with significant role in tissue regeneration, modulation of inflammatory response and cancer growth. Particularly, the MSC secretome consisting of soluble factors and EVs is increasingly recognized to mediate the therapeutic efficacy of MSCs [145, 146]. MSC-derived EVs have been shown to modulate cancer growth, metastasis, angiogenesis, immune response and resistance to chemotherapy and radiotherapy [147].

In CML, the MSC-derived EVs have been shown to increase sensitivity of CML cells to imatinib. EVs derived from human umbilical cord MSC potentiated the effects of apoptosis induced by imatinib in K562 cells [148, 149]. This could be explained by influencing intracellular signaling pathway in recipient cells by EVs. Possible mechanism could be transport of miRNA (such as miR-146a-5p) by vesicles as was described in a following study. Patients with CML, especially patients with imatinib-resistant CML with poor prognosis showed elevated levels of ubiquitin specific peptidase 6 (USP6) which inhibits glutaminase-1 (GLS1) ubiquitination leading to suppression of imatinib-induced apoptosis. EVs derived from human umbilical cord MSC were able to transfer miR-146a-5p to imatinib resistant K562 cells, downregulate USP6 levels, which was followed by induction of apoptosis [149]. In addition, expression of BAX was increased and expression of BCL-2 was decreased when K562 cells were treated together with imatinib and MSC-derived EVs [148]. Results from these studies combining MSC-derived EVs with imatinib suggest future potential therapeutic strategy to overcome the imatinib resistance and highlight the role of vesicle content in regulation of cellular pathways and signaling.

### Clinical translation and trials

Despite preclinical evidence underscoring the therapeutic and diagnostic promise of EVs in CML, clinical translation remains elusive. International clinical trial registries report only a limited number of specific entries focused on EVs applications in hematological malignancies. Notable example includes clinical trial No. NCT06245746, evaluating the efficacy and safety of umbilical cord MSCs-derived small EVs for consolidation chemotherapy-induced myelosuppression in acute myeloid leukemia patient post-complete remission (https://clinicaltrials.gov/study/NCT06245746). No dedicated clinical trials targeting EV-based diagnostics or treatments for CML appear in international registries.

### Future directions

While our understanding of the role of EVs in the BM microenvironment has advanced significantly, most findings have been derived from simplified in vitro models. Consequently, further research using more complex systems is required to clarify the precise functions of EVs in CML progression and drug resistance. Nonetheless, despite over a decade of intense scientific focus, EV research still faces several technical and methodological challenges. These include the lack of standardized protocols for vesicle isolation and storage, as well as inconsistent nomenclature across studies - issues that persist despite the efforts of the International Society for Extracellular Vesicles to establish the Minimal Information for Studies of Extracellular Vesicles (MISEV) guidelines [29, 36]. Moreover, technological improvements are needed to obtain high-purity EVs in sufficient quantities, particularly for applications in CML treatment. Multicenter validation studies using standardized protocols will be essential for progression into clinical applications. Clearly, further extensive research will be essential to overcome current limitations and enable practical application of EVs in clinical medicine.

## Conclusion

Over the past decade, EVs are extensively studied in cell-to-cell communication. Increasing numbers of studies are now investigating the roles of EVs in disease pathogenesis, including CML. EVs derived from CML leukemic cells have been shown to mediate communication with various cell types in the bone marrow microenvironment - such as endothelial cells, MSCs, immune cells, and osteoblasts - thereby influencing key pathophysiological processes, including the proliferation and survival of leukemic cells, neoangiogenesis, immune evasion, and therapy resistance. Leukemia-derived EVs also support leukemic cell proliferation and growth via secretion of vesicles with modified molecular content.

Molecules identified within EVs and implicated in these effects include among others various miRNAs (e.g., miR-126, miR-92, miR-21, miR-365), proteins such as amphiregulin, and cytokines like interleukin-8 (IL-8). EVs have been shown to impact multiple signaling pathways in recipient cells, including EGFR, ERK, AKT, NF-κB, and Src. Furthermore, they were shown to modulate the expression of MMP-9, CD44, apoptosis-regulating proteins, and nitric oxide (NO) production.

Accessibility of EVs from body fluids and minimally invasive approach to collect them demonstrates the potential of EVs as accessible diagnostic and prognostic markers of various diseases. Since the vesicle cargo reflects both physiological and pathological states of their cell of origin, analyzing the molecular content of EVs, such as proteins, miRNAs, and DNA including mutations, can offer valuable insights into treatment response or leukemia progression. This information could help in personalized medicine.

Additionally, small EVs, owing to their stability and biocompatibility, have emerged as promising delivery vehicles for therapeutic agents, including drugs and nucleic acids. EVs can be engineered to display specific surface molecules or to carry therapeutic cargo, enabling targeted delivery with minimal off-target effects. This strategy has been demonstrated to be feasible, for instance, in the delivery of BCR-ABL siRNA and imatinib to resistant leukemic cells. The rapidly evolving field of EVs research holds promise for the development of novel therapeutic strategies. To conclude, EVs from CML cells offer substantial potential to advance precision diagnostics, improve treatment-free remission monitoring, and enhance therapeutic strategies for patients with CML.

## Data Availability

No datasets were generated or analysed during the current study.

## Abbreviations

ABCG2: ATP-binding cassette super-family G member 2
ABL: Abelson leukemia virus oncogene
AKT: protein kinase B
AREG: amphiregulin
BAX: bcl-2-like protein 4
BCL-2: B-cell lymphoma 2
BCR: breakpoint cluster region gene
BM: bone marrow
BM-MSCs: bone marrow mesenchymal stromal cells
CML: chronic myeloid leukemia
EGFR: epidermal growth factor receptor
ERK: extracellular signal-regulated kinase
EVs: extracellular vesicles
HSCs: hematopoietic stem cells
HUVEC: human umbilical vein endothelial cells
IFITM3: interferon-induced transmembrane protein 3
IL-8: interleukin 8
LAMP2B: lysosome-associated membrane protein 2
LSC: leukemic stem cells
M-MDSC: monocytic myeloid-derived suppressor cells
MMP-9: matrix metalloproteinase-9
MSCs: mesenchymal stromal cells
NO: nitric oxide
PTEN: phosphatase and tensin homolog
ROS: reactive oxygen species
SENP2: sentrin-specific protease 2
TGF-β1: transforming growth factor beta 1
TKI: tyrosine kinase inhibitor
USP28: ubiquitin specific peptidase 28
USP6: ubiquitin specific peptidase 6
VCAM-1: vascular cell adhesion molecule 1
